# Dynamics of On-Off Neural Firing Patterns and Stochastic Effects near a Sub-Critical Hopf Bifurcation

**DOI:** 10.1371/journal.pone.0121028

**Published:** 2015-04-13

**Authors:** Gu Huaguang, Zhao Zhiguo, Jia Bing, Chen Shenggen

**Affiliations:** 1 School of Aerospace Engineering and Applied Mechanics, Tongji University, Shanghai 200092, China; 2 Centre for Computational Systems Biology, Fudan University, Shanghai 200433, China; Georgia State University, UNITED STATES

## Abstract

On-off firing patterns, in which repetition of clusters of spikes are interspersed with epochs of subthreshold oscillations or quiescent states, have been observed in various nervous systems, but the dynamics of this event remain unclear. Here, we report that on-off firing patterns observed in three experimental models (rat sciatic nerve subject to chronic constrictive injury, rat CA1 pyramidal neuron, and rabbit blood pressure baroreceptor) appeared as an alternation between quiescent state and burst containing multiple period-1 spikes over time. Burst and quiescent state had various durations. The interspike interval (ISI) series of on-off firing pattern was suggested as stochastic using nonlinear prediction and autocorrelation function. The resting state was changed to a period-1 firing pattern via on-off firing pattern as the potassium concentration, static pressure, or depolarization current was changed. During the changing process, the burst duration of on-off firing pattern increased and the duration of the quiescent state decreased. Bistability of a limit cycle corresponding to period-1 firing and a focus corresponding to resting state was simulated near a sub-critical Hopf bifurcation point in the deterministic Morris—Lecar (ML) model. In the stochastic ML model, noise-induced transitions between the coexisting regimes formed an on-off firing pattern, which closely matched that observed in the experiment. In addition, noise-induced exponential change in the escape rate from the focus, and noise-induced coherence resonance were identified. The distinctions between the on-off firing pattern and stochastic firing patterns generated near three other types of bifurcations of equilibrium points, as well as other viewpoints on the dynamics of on-off firing pattern, are discussed. The results not only identify the on-off firing pattern as noise-induced stochastic firing pattern near a sub-critical Hopf bifurcation point, but also offer practical indicators to discriminate bifurcation types and neural excitability types.

## Introduction

Identification of nonlinear dynamics in a single neuron or within a neuronal network is used to study neural coding mechanisms and integrated behaviors of a nervous system [1–4], and enables the depiction of complex oscillation patterns, such as periodic, chaotic or stochastic firing patterns [5–12]. Firing patterns often develop sequences of bifurcations in response to continuously changing biological parameters. For example, period-doubling cascade to chaos, which is a well-known transition, was observed in earlier experimental and theoretical studies of cardiac myocytes and nervous systems [9–12]. In addition to the bifurcations between firing patterns, four types of bifurcations ranging from resting state to firing pattern such as saddle-node bifurcation on an invariant cycle, a saddle-node bifurcation of an equilibrium point, sub-critical Hopf bifurcation, and super-critical Hopf bifurcation have been identified.

In the nervous system, stochastic resonance (SR) and coherence resonance (CR) always appear near the bifurcation points [13–25]. Nonlinear systems may display SR when their responses to both a weak periodic force and noise reach the maximum at a certain noise intensity level [13, 14]. The order of a system without an external periodic signal can also peak in response to noise intensity, showing CR effects [13, 15–17]. Near a saddle-node bifurcation on an invariant cycle and near a saddle-node bifurcation of the equilibrium point, noise-induced stochastic firing patterns manifest a continuous distribution in the interspike interval (ISI) histogram (ISIH) [18–20]. Near a supercritical Hopf bifurcation point, an integer multiple firing pattern of which the ISIs exhibit a discrete distribution in ISIH and are approximate integer multiples of a basic ISI has been found to be induced by noise [21–24]. These stochastic firing patterns were found to be between period-1 firing and resting states as some biological parameters were changed [18–24]. However, characteristics of the stochastic firing pattern simulated near a sub-critical Hopf bifurcation point remain unclear [25].

A firing pattern in which periodic repetitions of bursts containing spikes with roughly the same ISI value are interspersed with epochs of subthreshold oscillations (intracellular recording) or quiescent states (extracellular recording) has been frequently observed in experiments with different neural tissues, including GABAergic neurons in the nucleus basalis and the medial septum, neocortical neurons, mitral cells in the olfactory bulb, dorsal column nuclei neurons, magnocellular neurons in rodent hypothalamus trigeminal neurons, rat mesencephalic V neurons, entorhinal cortex layer II neurons, cortical layer 4 neurons, fast-spiking inhibitory interneurons in the somatosensory cortex, rat sciatic nerves after chronic constriction injury (CCI), and injured dorsal root ganglia neurons (CCD) [26–56]. The quiescent states pause the firing, resulting in an on-and-off type of firing pattern. For convenience, this firing pattern is referred to as on-off firing pattern in the present paper.

Studies of the on-off firing pattern have mainly focused on the ionic channels and physiological significance. For example, the on-off firing pattern has been suggested to play important roles in the generation of theta rhythms in the hippocampus and neocortex, and to participate in pathological pain in the CCD and CCI models [26–29]. A few investigations on the dynamics of the on-off firing pattern suggested it to be type III (elliptic) bursting or sub-Hopf/fold cycle bursting [30, 57]. However, no evidence has suggested that the type III bursting or sub-Hopf/fold cycle is changed to a resting state when a parameter is changed [30, 57]. In addition, because the recording time of the on-off firing pattern was short in most previous investigations, the dynamics of the on-off firing pattern were uncertain.

An important characteristic of the on-off firing pattern is that it can be directly changed to the resting state when certain physiological parameters are changed. This implies that the on-off firing may be induced by noise near a bifurcation of equilibrium point. However, the on-off firing pattern manifests characteristics different from that of simulated stochastic firing patterns near three other types of bifurcation except for the sub-critical Hopf bifurcation. Despite investigations into CR or stochastic effects near a sub-critical Hopf bifurcation point [25, 58], the relationship between the on-off firing pattern and the stochastic firing pattern near a sub-critical Hopf bifurcation point has not yet been determined. With the current study, we aim to fill this knowledge gap.

Here, typical examples of the on-off firing pattern were recorded in a CCI model, blood pressure baroreceptor, and hippocampal CA1 neuron. The recording time of the on-off firing pattern was long enough to identify the dynamics. The duration of both the bursts and quiescent states varied, and distinctions between on-off firing patterns and integer multiple firing patterns were identified. The ISI series of the on-off firing pattern was identified as stochastic using a nonlinear prediction method and autocorrelation function. Changes of the on-off firing pattern and the relationship between resting state and period-1 firing were also identified by adjusting physiological parameters. In the stochastic ML model, noise induced on-off firing pattern closely matched that observed in the experiment. The behavior of the on-off firing pattern was identified as noise-induced transitions between coexisting regimes near the sub-critical Hopf bifurcation point. In addition, the noise-induced escape rate from the focus and CR effect of the on-off firing pattern were quantified. Distinctions between the on-off firing pattern and noise-induced stochastic firing patterns near three other types of bifurcations of equilibrium points, and other viewpoints on the dynamics of on-off firing patterns are also discussed.

## Materials and Methods

### Ethics statement

All rats and rabbits were treated in strict accordance with institutional protocols. All experiments were approved by the University Biomedical Research Ethics Committee. Surgery was performed under general anesthesia using sodium pentobarbital (40 mg/kg, i.p.) in some rats, and using urethane (1 g/kg, i.v.) in rabbits and the remaining rats (1.5 mg/kg, i.p.). All efforts were made to minimize suffering.

### Experimental models

Bennet and Xie developed the CCI model, which reproduces many features of neuropathic pain disorders used to study neuropathic pain [38, 59]. Electrophysiological recordings from myelinated primary afferent axons revealed spontaneous impulsive activity originating at the nerve site, which led to abnormal spontaneous pain and contributed to maintenance of allodynia and hyperalgesia. In a series of previous investigations, the CCI model was used to study bifurcations and chaos of spontaneous neural firing patterns. In this context, the model was referred to as an experimental neural pacemaker, resembling a single neuron [5–8, 19–22]. This model was used for the current study.

Peripheral mononeuropathy was produced in adult male Sprague-Dawley rats (200–300 g) by loosely ligating the sciatic nerve following a method previously described. Under pentobarbital sodium anesthesia (40 mg/kg, i.p.; supplemented as necessary), the sciatic nerve was exposed unilaterally at the mid-thigh level by blunt dissection of the biceps femoris muscle, and then four loosely constrictive ligatures were tied around the nerve. The muscle and skin were closed in layers. After 8–12 days, the surgical field was exposed again and the site of injury was disconnected from the receptive field. Spike trains of spontaneous firing of individual fibers were recorded with a platinum wire electrode, placed approximately 2–3 cm proximal to the injured site at one end, and connected to PowerLab systems (ADInstruments, Australia) at the other. Meanwhile, spike trains were monitored with the PowerLab system during the experiment to assure that the recording was from a single unit. The sampling frequency was 10.0 kHz, and intervals between maximal values of successive spikes were detected seriatim as ISI series. Extracellular potassium ion concentration ([K^+^]_o_) was adjusted and changes in firing patterns were observed. More description of the model can be found in the Reference [5].

Male New Zealand White rabbits (2.0–3.0 kg) were anesthetized with urethane (1 g/kg, i.v.; supplemented as necessary). The trachea was intubated, and the rabbit was given room air. The right common carotid, innominate, and right subclavian arteries were exposed through a midcervical and midsternal incision. The right subclavian artery was tied proximal to the roots of the vertebral and internal thoracic arteries. Polyethylene tubing was cannulated into the right common carotid artery. Right common carotid pressure was detected via a hydraulic transducer connected to the cannulated tubing by a three-way valve. After the innominate artery was tied at its root, water sealing of the right common carotid artery was confirmed for a completely watertight cavity.

The right aortic depressor nerve was identified, separated, and cut to approximately 2 cm in length. The distal nerve end was placed on a plastic electrode plate covered with white mineral oil, and teased into divided bundles under a dissecting microscope. A single unit discharge was recorded with the aid of a platinum wire electrode. The single unit discharge signal and the pressure signal were simultaneously recorded with a PowerLab system. The time intervals between the maximal values of the successive spikes were recorded seriatim as ISI series. In the procedure, the static pressure within the sealed arterial lumen correlated directly with the change in the injected perfusion fluid volume.

Sprague-Dawley rats (approximately 3 weeks of age) were anesthetized with urethane (1.5 mg/kg, i.p.; supplemented as necessary). The brains were rapidly removed and transferred to ice-cold artificial cerebrospinal fluid (ACSF). Coronal slices (300 μm thick) were cut at 4°C using a vibratome (NatureGene, U.S.) and incubated in room-temperature ACSF for at least 1 h. In most cases, patch-clamp recordings were conducted within 6 h of tissue preparation. If slices or neurons appeared damaged (e.g., slices lost original shape or texture; neurons became granulated or with ill-defined margins), they were discarded.

For whole-cell patch-clamp recordings, slices were transferred to the recording chamber mounted on an upright microscope DM LFSA (Leica, Germany) and perfused with room-temperature ACSF at a rate of 1 mL/min. Hippocampal CA1 pyramidal neurons were visually selected by infrared DIC-video microscopy using a high-performance vidicon camera (Dage-MTI, U.S.). The microelectrode resistance was 3–5 MΩ. The electronic signal of the CA1 neuron was amplified using a MultiClamp 700B amplifier (Axon, U.S.) and digitized using a Digidata 1440A digitizer (Axon). Data were acquired and analyzed using pCLAMP 10 software (Axon). The time intervals between the maximal values of the successive spikes were detected seriatim as an ISI series. The current applied to the CA1 neuron via the microelectrode was adjusted.

### Theoretical model

The ML model represents an electrical circuit equivalent to a cellular membrane crossed by different currents, i.e., the voltage-gated calcium (Ca^2+^) current, the voltage-gated delayed-rectifier potassium (K^+^) current, and the leakage current [60]. The ML model is regarded as a canonical prototype for widely encountered types of bifurcations from the resting state to firing, which is defined as follows [58, 61]:

$$C\frac{dV}{dt}=-g_{Ca}m_{\infty }(V-V_{Ca})-g_{K}w(V-V_{K})-g_{L}(V-V_{L})+I$$ (1)

$$\frac{dw}{dt}=\phi \frac{\left[w_{\infty }-w\right]}{\tau_{w}}$$ (2)

Here, *t* is the time. The dependent variables are *V* (the membrane potential) and *w* (the activation of delayed rectifier K^+^ current). *I* is a DC current representing background ions. $m_{\infty }=0.5[1+\tanh(\frac{V-V_{1}}{V_{2}})]$, $w_{\infty }=0.5[1+tanh\frac{V-V_{3}}{V_{4}}]$,$\tau_{w}=1/\cosh(\frac{V-V_{3}}{2V_{4}})$. *C* = 20.0 μF/cm^2^, *V*_*Ca*_ = 120 mV, *g*_*K*_ = 8 mS/cm^2^, *g*_*L*_ = 2 mS/cm^2^, *V*_*L*_ = -60 mV, *V*_*1*_ = -1.2 mV, and *V*_*2*_ = 18 mV. The ML model manifests sub-critical Hopf bifurcation when *ϕ* = 0.04, *g_Ca_* = 4.4 mS/cm^2^, *V*_*3*_ = 2.0 mV, and *V*_*4*_ = 30 mV.

With the exception of thermal fluctuations of channel molecules, the most likely sources of noise are different between the three experimental models in this paper, as they consisted of systems with disparate functionality, cell morphologies, and network topologies. The main source of noise is fluctuations of membrane potential induced by modulations of calcium concentration for the neural pacemaker, by pressure for the depressor nerve, and by synaptic noise for the CA1 neuron. These fluctuations are consistent with a Gaussian white noise (ξ(*t*)) added to the right-hand side of the first equation, which forms the stochastic ML model. The statistical properties of ξ(*t*) are <ξ(*t*)> = 0 and <ξ(*t*)ξ(*t'*)> = *2Dδ*(*t-t*'), where *D* is the noise intensity, and *δ*(·) is the Dirac *δ*-function. The unit of *D* is (μA)^2^/cm^4^. Different ways of incorporating channel noise into the Hodgkin-Huxley equations were discussed in [62]. The deterministic and stochastic ML models were solved using a Mannella numerical integration method [63] with an integration time step of 0.04 ms. An action potential was said to occur when *V* crossed a value of 25.0 mV from below.

To simulate experimental neural pacemakers in which [K^+^]_o_ was adjusted as the bifurcation parameter, the reversal potential of K^+^ in the ML model, *V*_*K*_, was chosen as the control parameter. The unit of current *I* in the ML model was μA/cm^2^ and *I* was fixed at 90.7 μA/cm^2^. To render depressor nerve experiments in which static blood pressure was adjusted as the bifurcation parameter and CA1 neuron experiments in which the depolarization current was adjusted as the bifurcation parameter compatible with each other, *I* was chosen as the control parameter and *V*_*K*_ was fixed at -84 mV in the ML model.

### Software packages used for bifurcation calculation

The bifurcation diagrams of ML model are constructed with AUTO as a component of the XPPAUT software. More detailed information about the XPPAUT software was described in a previous study [64].

### Time series analysis method

Many time series analysis methods, such as the recurrence method [65–68], Lyapunov exponent [69, 70], nonlinear prediction or forecast [5, 8, 71, 72], and autocorrelation function [73–75] can be used to classify a time series as chaotic or stochastic. In the present investigation, normalized prediction error (*NPE*) and autocorrelation function are used.

The algorithm of the simple nearest-neighbor method to calculate *NPE* [5, 8, 71, 72] is as follows: choose a dimension *m*, a time series, *t*_*i*_ (*i* = 1,2,...,*L*), is transformed to *L-m*+1 state points in space *R* ^*m*^. For a point *V*_*n*_ = (*t*_*n*_,*t*_*n+1*_,...,*t*_*m+n+1*_)(*n* = 1,2,...,*L-m*+1) in space *R* ^*m*^, *M* = *b*(*L-m*+1) points nearest to *V*_*n*_ are chosen (*b*=1) and written as *U*_*k*_ = (*t*_*jk*_, *t*_*jk*+1_, …, *t*_*jk*+*m*_) (1 £ *k* £ *M*). The average $P_{n,h}=\frac{1}{M}\sum_{k=1}^{M}t_{j_{k}+h}$ is then used to approximate the future value *t*_*n+h*_. The difference *p*_*n*,*h*_-*t*_*n*,*h*_ is the *h*-th step prediction error for point *V*_*n*_. For all state points, the *NPE* is defined as follows:

$$NPE(h)=\frac{{\left(\frac{1}{L-m+1}\sum_{n=1}^{L-m+1}{\left(p_{n,h}-t_{n+h}\right)}^{2}\right)}^{\frac{1}{2}}}{{\left(\frac{1}{L-m+1}\sum_{n=1}^{L-m+1}{\left(\bar{t}-t_{n+h}\right)}^{2}\right)}^{\frac{1}{2}}}$$ (3)

Here, $\bar{t}$ is the average of the time series *t*_*i*_. By definition, *NPE* values far below 1.0 indicate that the time series is predictable beyond the baseline prediction of the series mean, and values nearly equaling 1.0 indicate that the time series is unpredictable. The predictable characteristic of a time series means that the time series should be chaotic (deterministic) as opposed to an unpredictable series that is stochastic.

In the previous studies, if *NPE* values of an ISI series approached 1.0 in both short-term and long-term predictions, the ISI series was considered stochastic [5, 8, 71, 72]. If the *NPE* value of an original ISI series was much less than 1.0 in short-term prediction and approached 1.0 in long-term prediction, but the *NPE* of the surrogate data was almost always equivalent to 1.0, the original and surrogate ISI series were considered as chaotic and stochastic [5, 8, 71, 72], respectively.

The autocorrelation function [73–75] of a time series *t*_*i*_ (*i* = 1, 2,…, *L*) is calculated as follows:$\rho (\tau )=\frac{<\bar{t_{i}t_{i+\tau }}>}{<{\bar{t_{i}}}^{2}>}$, where$\bar{t_{i}}=t_{i}-<t_{i}>$, <> is the average over time, and *τ* (*τ*<*L*) is time delay. Obviously, *ρ*(*τ*) = 1 when *τ = 0*. Theoretically, a time series is stochastic if *ρ*(*τ*) = 0 for all *τ>0*, and is chaotic or deterministic if *ρ*(*τ*)≠0 for at least one *τ*>0. In practice, all -0.05<*ρ*(*τ*)<0.05 were regarded as 0.

## Results

### Three kinds of firing patterns of CCI model under control condition

Under control condition with [K^+^]_o_ at 5 mmol/L, the period-1 firing pattern, the integer multiple firing pattern, and the on-off firing pattern were recorded from 16, 12, and 69 pacemakers, respectively, of 23 rats. The different individual pacemakers under the control condition presented different firing modes due to individual variation of the electrophysiological parameter configurations. The representative firing patterns recorded from different pacemakers were introduced as follows.

**Fig 1:**
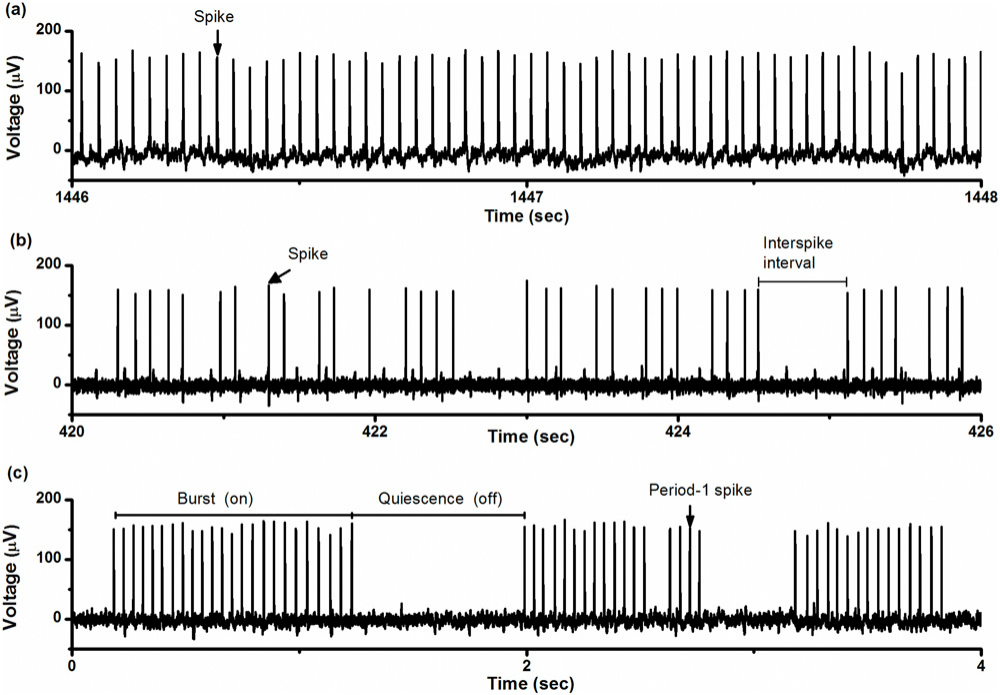
Firing patterns. Each pattern was recorded from a different experimental neural pacemaker under control condition with 5 mmol/L [K^+^]_o_. (a) Period-1 firing pattern; (b) integer multiple firing pattern; (c) on-off firing pattern.

The period-1 firing pattern manifested as spikes with nearly equal ISI values, as shown in Fig 1(a). The variations in ISIs, which were induced by internal noise of the pacemaker and recording error of the PowerLab system, were small. The coefficient of variability (CV, standard deviation/mean × 100%) of the ISI series, shown in Fig 1(a), was approximately 3.95%.

The integer multiple firing pattern and the on-off firing pattern are shown in Fig 1(b) and 1(c), respectively. The integer multiple firing pattern manifests random suppression of spikes from the period-1 firing pattern [21, 22] while the on-off firing pattern exhibits random suppression of clusters of spikes from the period-1 firing pattern. This results in alternations between bursts containing multiple spikes with nearly equal ISI values and quiescent states over time. To be distinguished from the concept of period-1 firing pattern, the spikes with nearly equal ISI values within the burst are called period-1 spikes in the present study.

### On-off firing pattern of CCI model

The characteristics of the on-off firing pattern are shown in Fig 1(c). ISIs of period-1 spikes within bursts had a small CV. The ISI value within bursts was 0.0450 ± 0.0037 s and CV was 8.27%. Most given bursts could contain many period-1 spikes (16.0 ± 10.9 spikes). The ratio of the number of spikes to the number of quiescent states (labeled as *R*_*sq*_) was 16, indicating that the number of spikes per burst was large. The duration of the individual quiescent states was 0.3540 ± 0.2095 s and the duration of the bursts was 0.6826 ± 0.4950 s. The CVs for quiescent states and bursts were 72.52% and 59.18%, respectively. The CV of ISIs of the on-off firing pattern was 95.46%.

**Fig 2:**
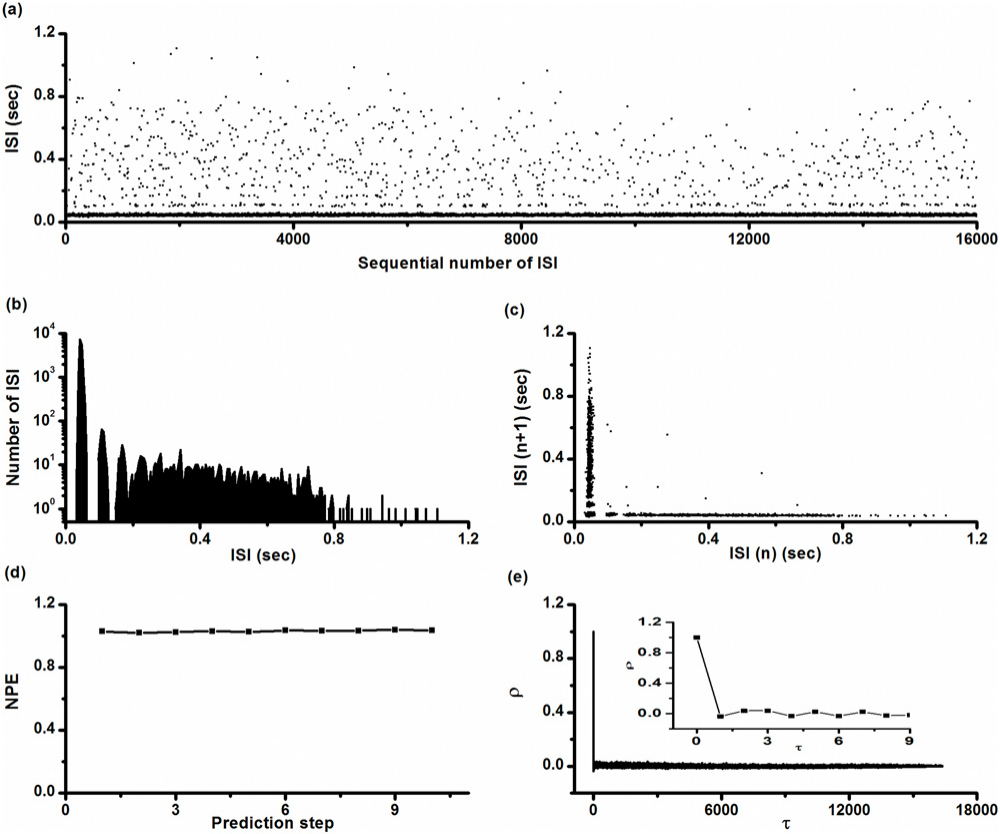
Characteristics of on-off firing pattern. (a) ISI series; (b) ISI histogram; (c) first return map of ISI series; (d) *NPE* of ISI series; (e) autocorrelation function of ISI series; Insert: autocorrelation function when *τ* < 10.

The ISI series and ISIH are shown in Fig 2(a) and 2(b), respectively. In ISIH, two discrete peaks located at shorter ISI values and other longer ISIs exhibited continuous distribution. Many points located within the lowest ISI layer are shown in Fig 2(a), and the first peak in ISIH shown in Fig 2(b) was extra high, consistent with multiple spikes per burst. The first return map of the ISI series was mainly composed of two groups of points, one group forming point clusters parallel to the x-coordinate and the other parallel to the y-coordinate, as shown in Fig 2(c). Except for these two groups, there were only 12 isolated points, indicating that the intra-burst spikes are repetitive and single spikes are infrequent. We counted 16,376 spikes in this typical example and observed only 12 single spikes.

*NPE* of ISI series shown in Fig 2(a) was calculated with *m* = 3–8 and *b* = 0.5%, 1%, and 2%. *NPE* values were found to be independent of *m* and *b*. For example, when *m* = 4 and *b* = 1%, *NPE* was nearly 1.0 when predicted from step 1 to step 9, as shown in Fig 2(d), indicating that such firing patterns may result from stochastic dynamics. The values of *ρ*(*τ*) of this ISI series nearly equaled 0 (-0.035<*ρ*(*τ*)<0.039) for all *τ*>0, as shown in Fig 2(e), also indicating that the on-off firing pattern was stochastic.

**Fig 3:**
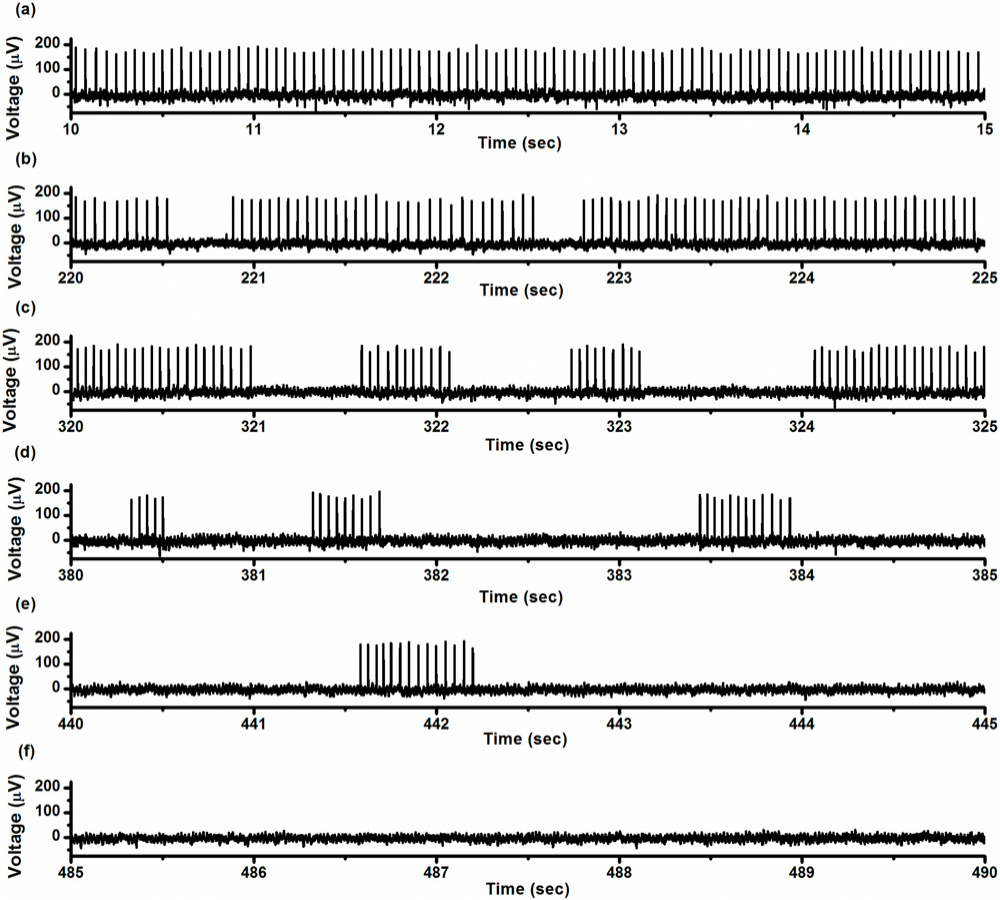
Electrical activity patterns for the CCI experiments. Period-1 firing pattern to resting state via on-off firing pattern during a changing process induced by decreasing [K^+^]_o_ from 10 mmol/L to zero. (a) Period-1 firing pattern at 10–15 s; (b) on-off firing pattern at 220–225 s; (c) on-off firing pattern at 320–325 s; (d) on-off firing pattern at 380–385 s; (e) on-off firing pattern at 440–445 s; (f) resting state at 485–490 s.

To confirm dynamics of such on-off firing patterns, we changed the physiological parameters to track firing pattern evolution. Decreasing [K^+^]_o_ from 10 mmol/L to 0 mmol/L, the period-1 firing (Fig 3(a)) was changed into the on-off firing pattern (Fig 3(b)-3(e)) at first and then into a resting state (Fig 3(f)). During this process, the duration of the burst decreased and the duration of the quiescent state increased.

**Fig 4:**
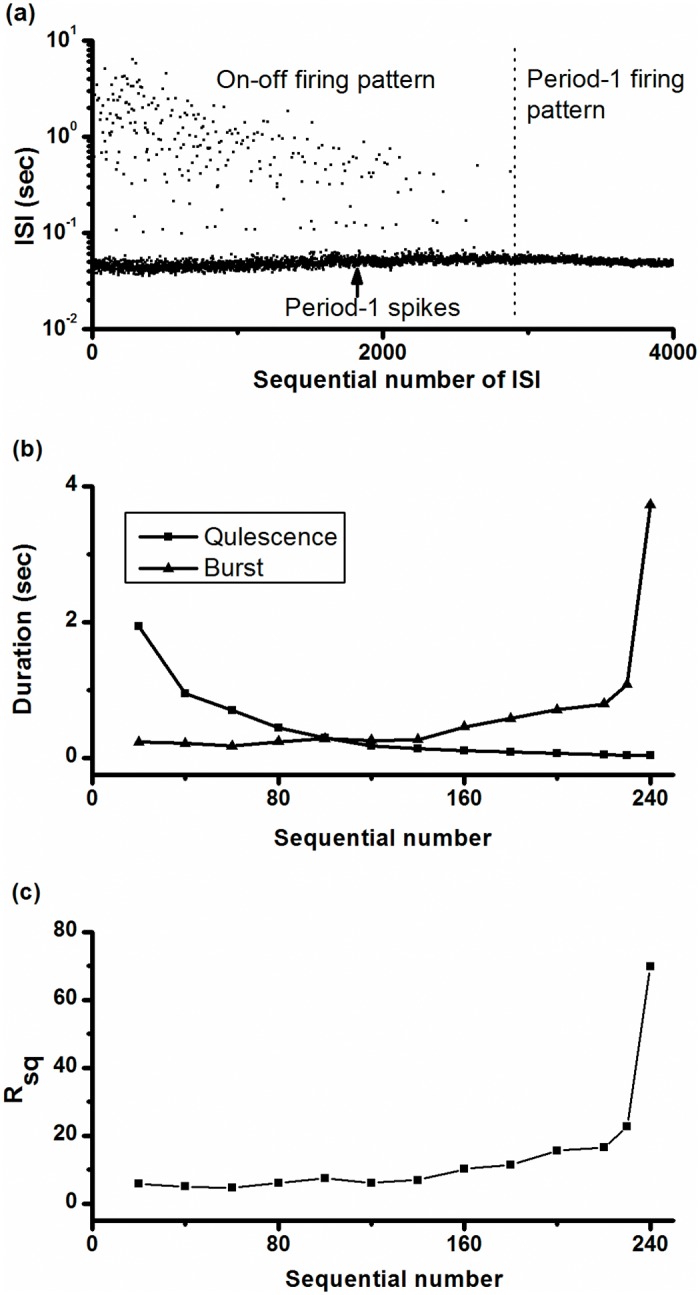
Changes in the on-off firing pattern. (a) ISI series of on-off firing pattern (from 1 to 2,900, left to the dotted line) changed to period-1 firing pattern (from 2,901 to 4,000, right to the dotted line) as [K^+^]_o_ increased from zero to 10 mmol/L; (b) mean duration of quiescent state (line with square) and burst (line with triangle) of on-off firing pattern with respect to sequential number; (c) ratio of number of spikes to the number of quiescent states (*R*_*sq*_) with respect to sequential number of quiescent states.

Increasing [K^+^]_o_ from 0 mmol/L to 10 mmol/L induced a transition from resting state to the on-off firing pattern, and then to the period-1 firing pattern, as shown in Fig 4(a). The on-off firing pattern and the period-1 firing pattern are located on left and right sides of the dotted line (sequential number of ISI is 2900), respectively. During the transition process, the burst duration of the on-off firing pattern lengthened and the duration of the quiescent state shortened, as shown in Fig 4(b). The *R*_*sq*_ increased from approximately 4 to approximately 60, as shown in Fig 4(c). These results indicated that the on-off firing was an intermediate behavior between the resting state and period-1 firing pattern as the physiological parameter [K^+^]_o_ was changed.

### On-off firing pattern observed from the baroreceptor

**Fig 5:**
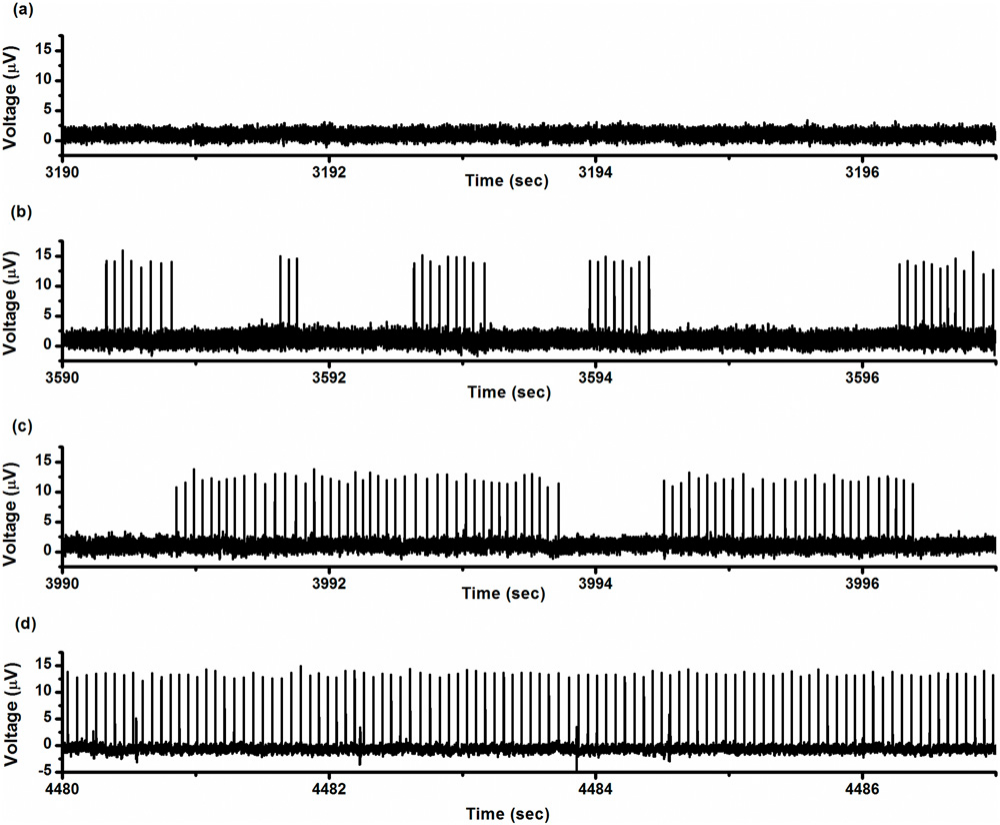
Electrical activity patterns observed from a depressor nerve. Resting state was changed to period-1 firing pattern as static pressure (SP) was increased. (a) Resting state at SP = 57.41 mmHg; (b) on-off firing pattern at SP = 69.40 mmHg; (c) on-off firing pattern at SP = 85.77 mmHg; (d) period-1 firing pattern at SP = 101.08 mmHg.

On-off firing patterns were recorded from 12 depressor nerve fibers. One example is shown in Fig 5. The resting state was recorded when the static pressure was 57.41 mmHg (Fig 5(a)). When the static pressure was 69.40 mmHg, the on-off firing pattern was recorded (Fig 5(b)). When the static pressure was 85.77 mmHg, won-off firing pattern was still observed (Fig 5(c)). As the static pressure was increased to 101.08 mmHg, the firing pattern changed to a period-1 firing pattern (Fig 5(d)). The on-off firing was between the resting state and period-1 firing pattern as the static pressure was changed. The characteristics and changes of the on-off firing pattern were similar to those observed in the neural pacemaker.

### On-off firing pattern observed from CA1 pyramidal neuron

**Fig 6:**
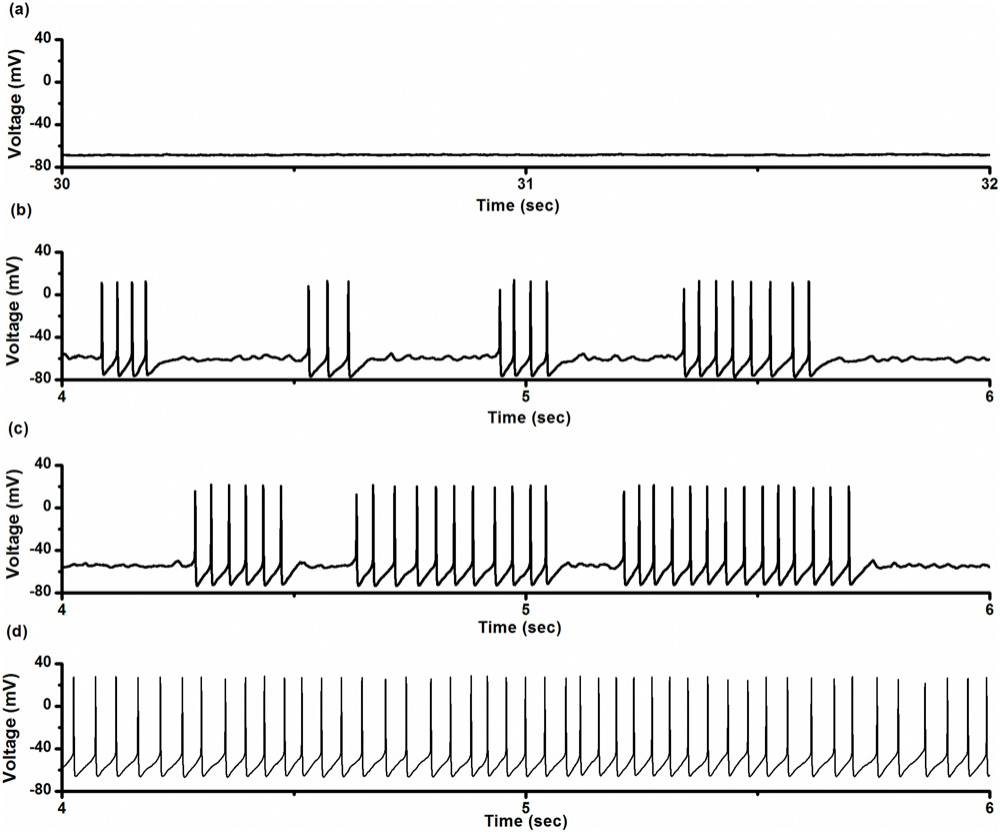
Electrical activity patterns recorded from a CA1 neuron. Resting state was changed to period-1 firing pattern via on-off firing patterns as depolarization current was increased. (a) Resting state under control conditions (no depolarizing current); (b) on-off firing pattern when depolarization current was 15 pA; (c) on-off firing pattern when depolarization current was 25 pA; (d) period-1 firing pattern when depolarization current was 40 pA.

On-off firing patterns were recorded from 8 CA1 neurons. One example recorded from a CA1 neuron is shown in Fig 6. The resting state was recorded under control conditions with zero depolarization current (Fig 6(a)). When a depolarization current of 15 pA was applied to the neuron, the on-off firing pattern was recorded (Fig 6(b)). When the depolarization current was 25 pA, the neuron still showed an on-off firing pattern (Fig 6(c)). As the depolarization current was increased to and fixed at 40 pA, the firing pattern changed to the period-1 firing pattern (Fig 6(d)). The characteristics and the changes in the on-off firing pattern resembled those observed in the experiments on the neural pacemaker and depressor nerve.

### Distinction of the integer multiple firing pattern

**Fig 7:**
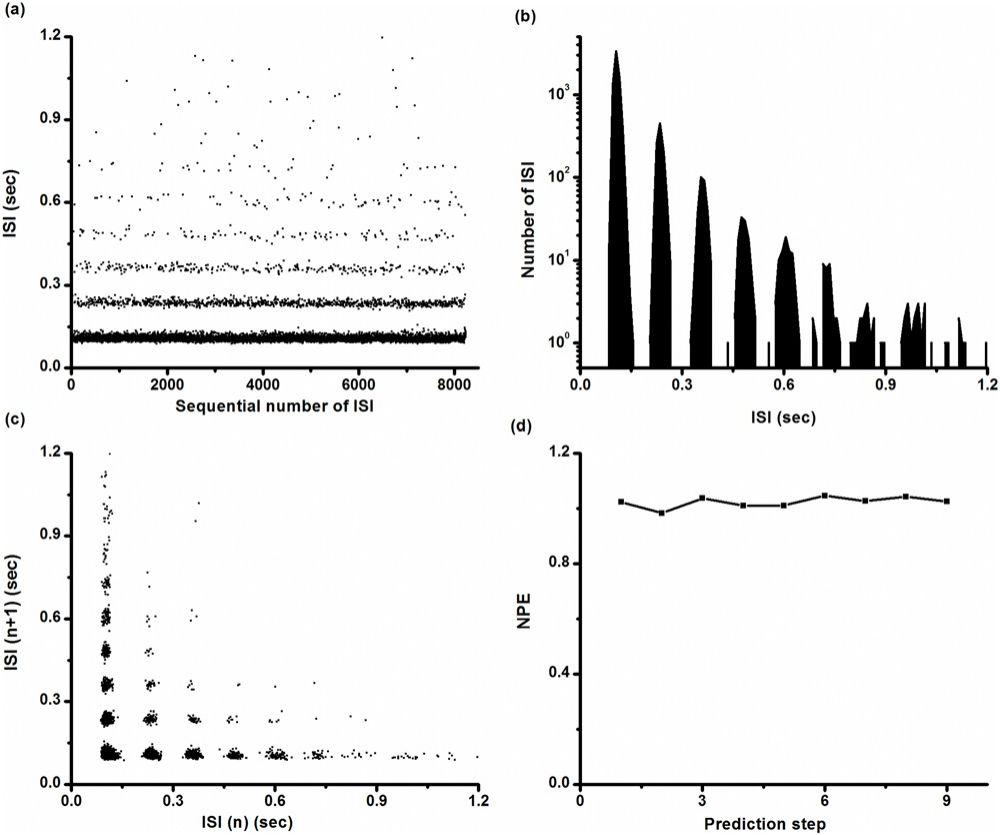
Characteristics of the integer multiple firing pattern. (a) ISI series; (b) ISI histogram; (c) first return map of ISI series; (d) *NPE* of the ISI series.

An example of the ISIs of the integer multiple firing pattern recorded from a neural pacemaker under control condition is shown in Fig 7. There were 8,231 spikes and 1,531 quiescent states, and the value of *R*_*sq*_ was 5.4, which is much less than that of the on-off firing pattern (16.0). The ISI series had multiple layers (Fig 7(a)) corresponding to multiple discrete peaks in ISIH (Fig 7(b)). The ISI values were approximately integer multiples of the ISI of the first peak, i.e., the basic ISI. The exponential decay pattern of peak amplitudes in the ISIH was investigated in a previous study [35]. In the first return map of ISI series, a lattice structure was observed (Fig 7(c)). Except for two bands of points parallel to the x- and y-coordinates 213 points were identified, suggesting that there were 213 single spikes out of 8,231 spikes. The ratio of single spikes to all spikes was 2.59% for the integer multiple firing pattern, which was greater than that of the on-off firing pattern (0.073%), wherein only 12 single spikes were observed out of 16,366 spikes. When *m* = 4 and *b* = 1%, *NPE* of the ISI series is nearly 1.0 when predicted from step 1 to step 9, as shown in Fig 7(d), implying that no deterministic structures were detected in the ISI series. The CV of ISIs of the integer multiple firing pattern was 86.12%.

**Fig 8:**
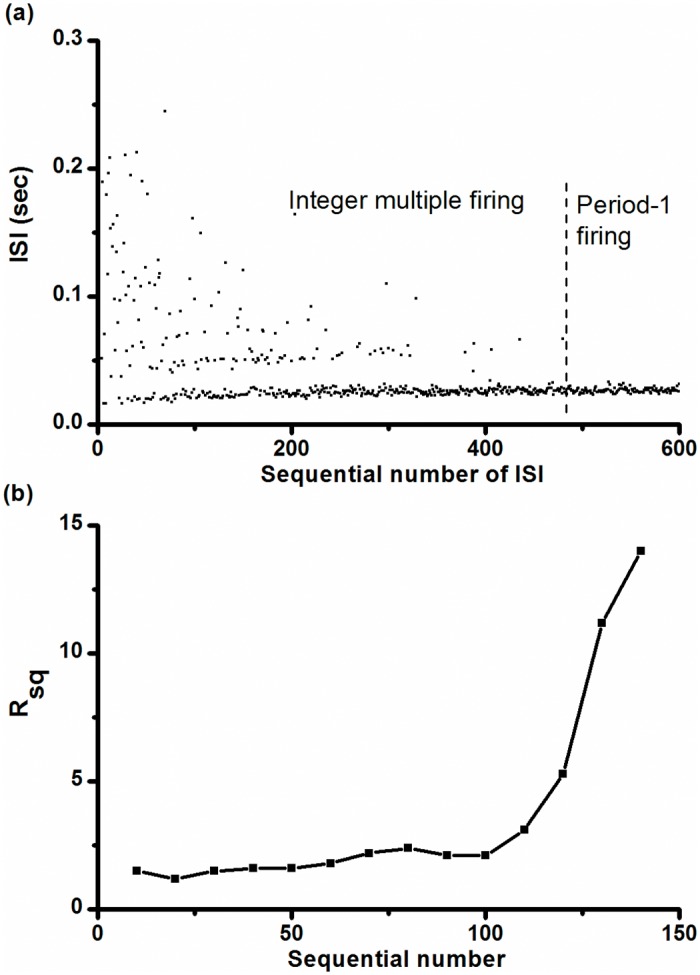
Changes in the integer multiple firing pattern for the CCI experiment. (a) ISI series of the integer multiple firing pattern (from 1 to 480, left to the dotted line) changed to the period-1 firing pattern (from 481 to 600, right to the dotted line) as [K^+^]_o_ increased from zero to 10 mmol/L; (b) ratio of number of spikes to the number of quiescent states (*R*_*sq*_); each data point was calculated from 10 contiguous quiescent states.

As [K^+^]_o_ was increased in a pacemaker, the integer multiple firing pattern (left to the vertical dashed line) was changed to the period-1 firing pattern (right to the vertical dashed line), and the changes of ISIs are shown in Fig 8(a). The value of *R*_*sq*_ shown in Fig 8(b) increased from approximately 1 to approximately 14, which is less than that of the on-off firing pattern shown in Fig 4(c).

The integer multiple firing was simulated near a super-critical Hopf bifurcation point in the stochastic Chay model [22]. This suggested that the on-off firing pattern may be induced by noise near a sub-critical Hopf bifurcation point.

### Bifurcations in the deterministic ML model

**Fig 9:**
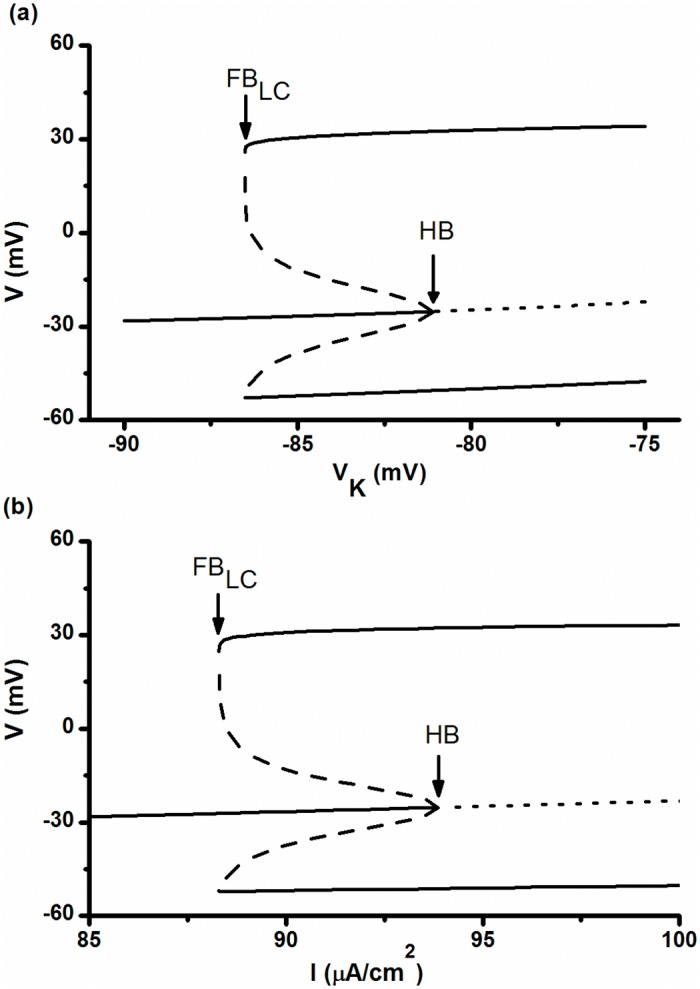
Bifurcation structure of the deterministic ML model. (a) Bifurcations with respect to *V*_*K*_ when *I* = 90.7 μA/cm^2^. (b) Bifurcations with respect to *I* when *V*_*K*_ = -84 mV.

In the deterministic ML model with *I* = 90.7 μA/cm^2^, *V*_*K*_ ≈-81.17 mV is a sub-critical Hopf bifurcation (HB) point of the equilibrium point, and *V*_*K*_ ≈-86.50 mV is a fold bifurcation of the limit cycle (FB_LC_), as shown in Fig 9(a). The HB and FB_LC_ points are labeled by arrows. When *V*_*K*_ < -86.50 mV, the ML model manifests a stable focus corresponding to the resting state. When *V*_*K*_ >-81.17 mV, a stable limit cycle corresponding to period-1 firing appears. When -86.50 mV < *V*_*K*_ < -81.17 mV, a stable focus, an unstable limit cycle, and a stable period-1 limit cycle coexist, as shown in Fig 9(a), which results in bistability. The upper (lower) thin solid line, the upper (lower) dashed line, and the bold solid line correspond to the maximal (minimal) amplitude of the stable period-1 limit cycle, the unstable limit cycle, and the stable focus, respectively.

In the deterministic ML model with *V*_*K*_ = -84 mV, the bifurcation structure with respect to *I* is shown in Fig 9(b), similar to that shown in Fig 9(a). There is a sub-critical Hopf bifurcation point when *I*≈ 93.86 μA/cm^2^ and a fold bifurcation of limit cycle when *I*≈ 88.29 μA/cm^2^. In the following subsection, bifurcation dynamics with respect to *V*_*K*_ were investigated as an example.

**Fig 10:**
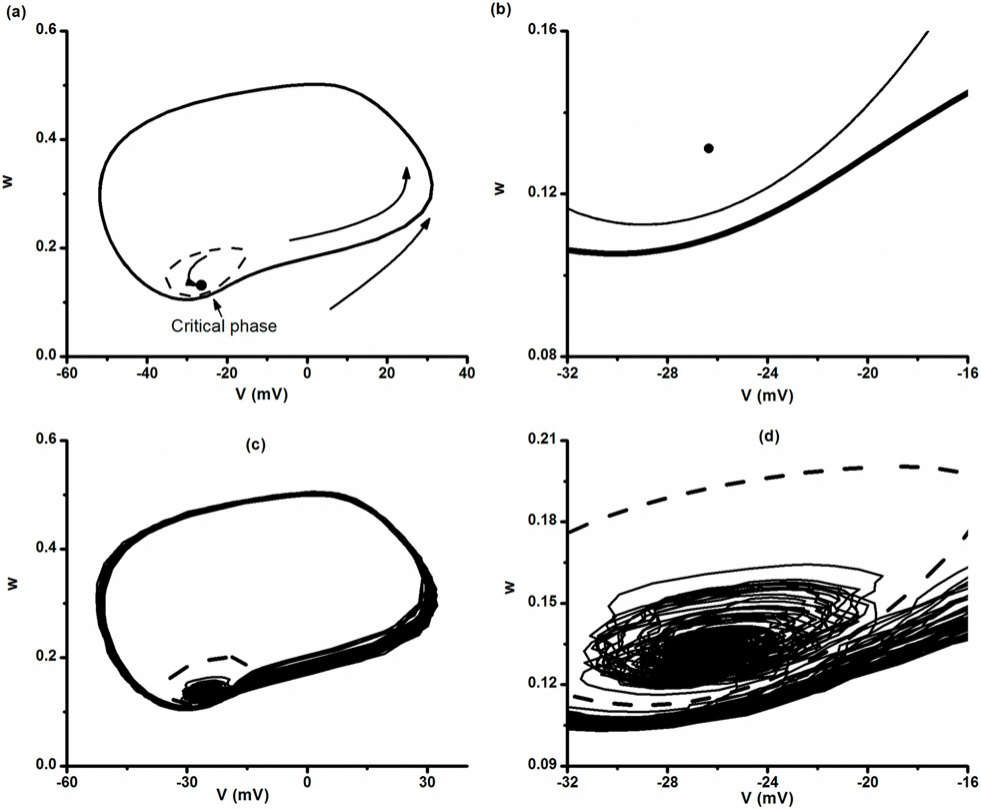
Trajectories in (*V*, *w*) plane in the ML model. *I* = 90.7 μA/cm^2^ and *V*_*K*_ = -84 mV. (a) The stable focus (bold dot), unstable limit cycle (dashed line), and stable period-1 limit cycle (solid bold line) in the deterministic model; evolution of the stable limit cycle is anti-clockwise; (b) enlargement of Fig 10(a) near the critical phase; (c) unstable limit cycle (dashed line) and the on-off firing pattern (solid line) in the stochastic ML model when *D* = 0.04 (μA)^2^/cm^4^; (d) enlargement of Fig 10(c) near the critical phase.

When *V*_*K*_ = -84 mV, the stable limit cycle, unstable limit cycle, and stable focus are presented by a bold solid line, a thin dotted line, and a circle point, respectively, in (*V*, *w*) plane, as shown in Fig 10(a). The unstable limit cycle separates the basins of attraction of the stable focus and the stable limit cycle. The evolution of the stable limit cycle is anti-clockwise. If the initial values of *V* and *w* are outside the unstable limit cycle, the system will evolve to and stay along the stable limit cycle. If the initial values of *V* and *w* are inside the unstable limit cycle, the system will evolve to the stable focus. The distance between the stable and unstable limit cycles is very short near a critical phase labeled by an arrow. The enlargement of the critical phase is shown in Fig 10(b).

### Speculation of stochastic effect on the bistable behavior

In the stochastic ML model adjusted to be within the parameter range of bistability, the noise with suitable intensity may induce transitions between the coexisting attractors. Because the focus, the stable limit cycle, and the unstable limit cycle are close near the critical phase shown in Fig 10(a) and 10(b), the transitions will likely happen near this phase. Therefore, the noise evokes transitions between a smaller ring and a bigger ring. The smaller ring corresponds to noise-induced subthreshold oscillations around the stable focus, whereas the larger ring to excursions around the stable limit cycle. The system evolves alternatively on the two rings to form a stochastic firing pattern alternating between burst with multiple period-1 spikes and quiescent states. These can be found in a simulation trial of on-off firing pattern in the stochastic ML model when *I* = 90.7 μA/cm^2^, *V*_*K*_ = -84 mV, and *D* = 0.04 (μA)^2^/cm^4^, as shown in Fig 10(c) and 10(d).

Because both of the coexisting attractors in the deterministic model are stable, the system appears to reside within the basin of attraction until it is forced to leave this basin, which results in long resident time along the limit cycle (burst duration) and along the subthreshold oscillation (duration of quiescent state). In addition, because the noise-induced transitions between the quiescent state and burst were stochastic, the duration of both quiescent states and bursts had large CVs. As illustrated in Fig 9(a), the basin of attraction of the stable focus decreases while that of the stable limit cycle increases with increasing *V*_*K*_ in the deterministic ML model. For this reason, in the stochastic ML model, it can be speculated that as *V*_*K*_ increases, the burst duration increases whereas the duration of quiescent state decreases.

### On-off firing pattern simulated in the stochastic ML model with respect to *V*_*K*_

**Fig 11:**
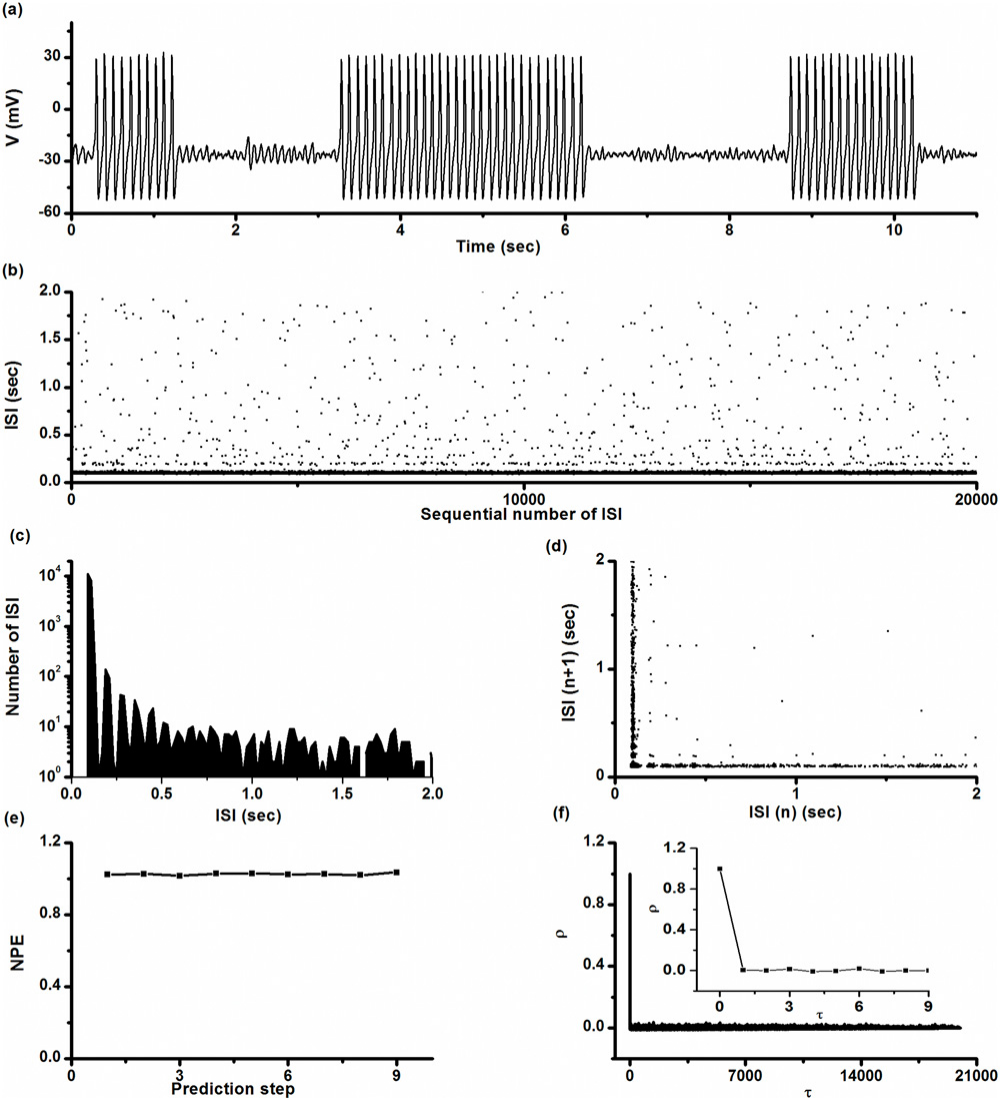
On-off firing pattern simulated in the stochastic ML model. *D* = 0.04 (μA)^2^/cm^4^, *I* = 90.7 μA/cm^2^, and *V*_*K*_ = -84 mV. (a) Firing trains; (b) ISI series; (c) ISI histogram; (d) first return map of ISI series; (e) *NPE* of ISI series; (f) autocorrelation function of ISI series; Insert: autocorrelation function when *τ* < 10.

As speculated, the long duration of both quiescent states and bursts, and their variations can be found using a simulation trial of the on-off firing pattern in the stochastic ML model with *I* = 90.7 μA/cm^2^, *V*_*K*_ = -84 mV, and *D* = 0.04 (μA)^2^/cm^4^. The firing trains are shown in Fig 11(a).

The simulated on-off firing was similar to experimental observations (Fig 11(a)-11(d)). In a typical trial, ISI of period-1 spikes within bursts was 100.50 ± 5.76 ms with a small CV of 5.75%. There were 21.4 ± 19.4 spikes per burst. A marked CV appeared in the duration of the quiescent state (115.58% from 1189.76 ± 1375.24 ms) and burst duration (92.56% from 2092.36 ± 1936.60 ms). In the first return map of ISI series, except for the two groups of points parallel to the x- or y-coordinates, respectively, there were 71 isolated points, indicating that the intra-burst spikes are repetitive and single spikes were infrequent. We counted 19,990 spikes in this typical trial and found only 71 single spikes.

The *NPE* of this ISI series was nearly 1.0 when predicted from step 1 to step 9, as shown in Fig 11(e), indicating that there were no deterministic structures within the ISI series. *ρ*(*τ*) of ISI series nearly equaled 0 (-0.01 < *ρ*(*τ*) < 0.014) for all *τ*>0, as shown in Fig 11(f). Both results indicated that the on-off firing pattern was stochastic.

**Fig 12:**
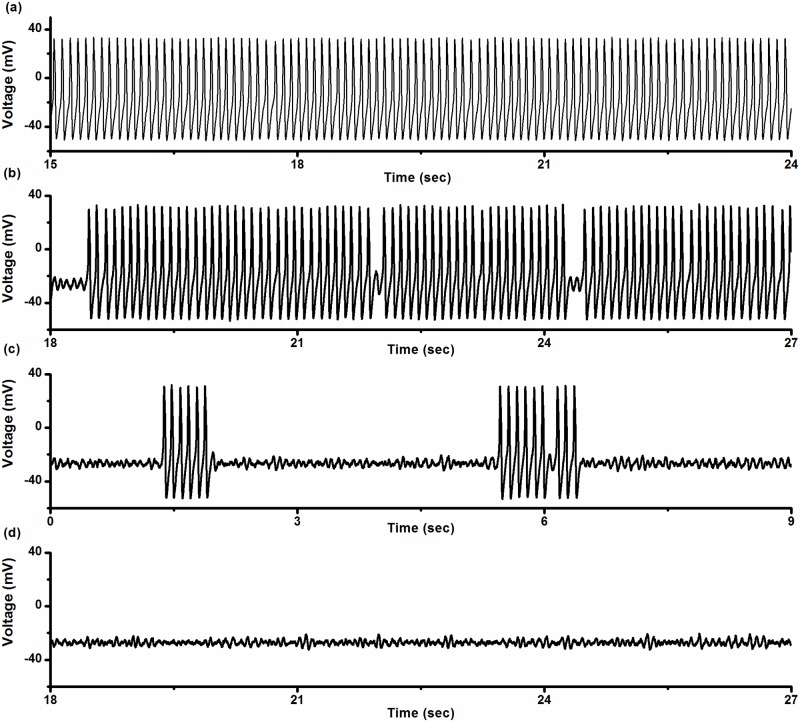
Electrical activity patterns simulated in the stochastic ML model. Period-1 firing pattern was changed into resting state via on-off firing pattern as *V*_*K*_ decreased (*D* = 0.04 (μA)^2^/cm^4^, *I* = 90.7 μA/cm^2^). (a) Period-1 firing pattern when *V*_*K*_ = -81 mV; (b) on-off firing pattern when *V*_*K*_ = -83.5 mV; (c) on-off firing pattern when *V*_*K*_ = -85 mV; (d) resting state when *V*_*K*_ = -87 mV.

In the stochastic ML model, the on-off firing pattern also appeared to alternate between quiescent states and bursts with period-1 spikes. For a given noise intensity, there was a parameter range of *V*_*K*_ in which such an on-off firing pattern could be simulated. For example, when *D* = 0.04 (μA)^2^/cm^4^, the parameter range for this on-off firing pattern was -86.8 mV ≤ *V*_*K*_ ≤ -80.92 mV. When *V*_*K*_ < -86.8 mV, the behavior of the stochastic ML model involved subthreshold oscillations around the stable focus. When *V*_*K*_ > -80.92 mV, the trajectories evolved along the stable limit cycle to form a period-1 firing pattern. Examples of the period-1 firing pattern (*V*_*K*_ = -81 mV), the on-off firing pattern (*V*_*K*_ = -83.5 mV and -85 mV), and resting state (*V*_*K*_ = -87 mV) were provided, as shown in Fig 12. The relationship between the on-off firing pattern and its neighboring firing patterns in parameter space were the same as that in the experiment.

**Fig 13:**
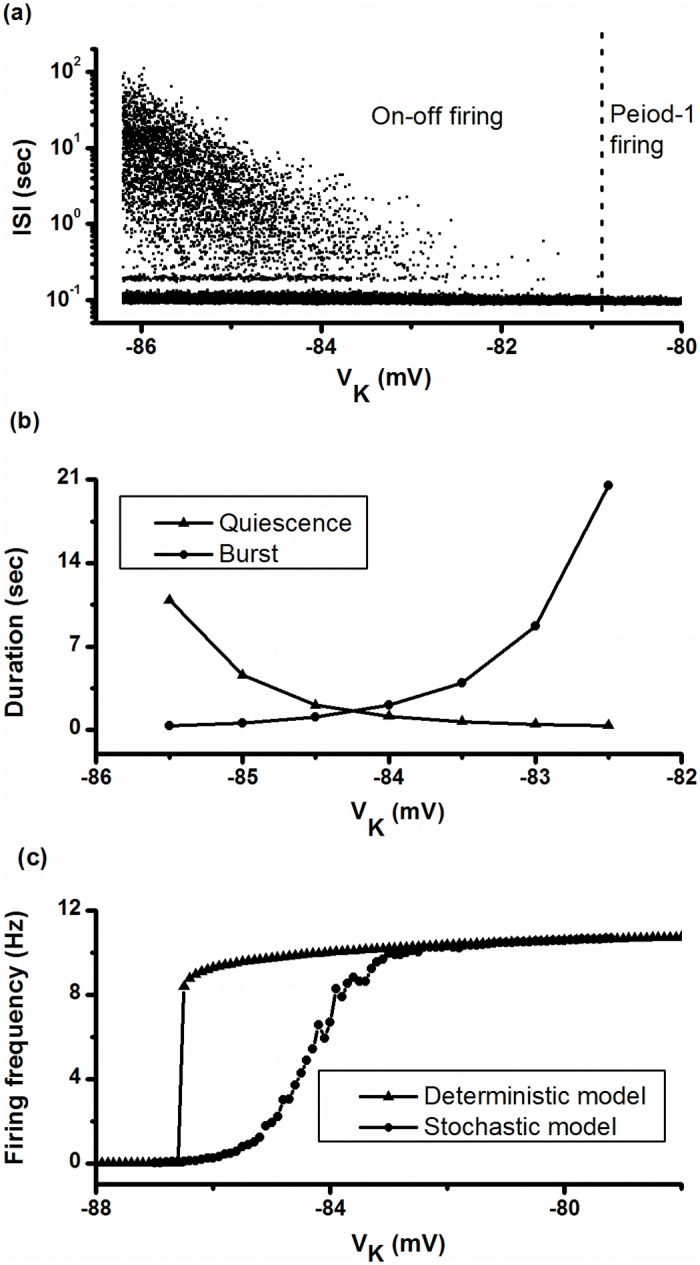
Changes of firing pattern with respect to *V*_K_ in the ML model. (a) On-off firing pattern (left to the dotted line) is changed to period-1 firing pattern (right to the dotted line) when *D* = 0.04 (μA)^2^/cm^4^; (b) changes in the duration of both quiescent state (triangle) and burst (circle) when *D* = 0.04 (μA)^2^/cm^4^; (c) changes in firing frequency when *D* = 0.04 (μA)^2^/cm^4^ (line with dots) and *D* = 0 (μA)^2^/cm^4^ (line with triangles).

The changes of ISIs from on-off firing patterns (left to the dotted line) to period-1 firing patterns (right to the dotted line), as obtained by increasing the control parameter *V*_*K*_, are shown in Fig 13(a) (-86.2 mV < *V*_*K*_ < -80 mV). As speculated, the duration of burst increased but the duration of the quiescent state decreased with respect to the increase in *V*_*K*_, as shown in Fig 13(b), and this closely matched the experimental observations. Correspondingly, the firing frequency gradually increased from a value nearly zero to a fixed value, as shown by the line with dots in Fig 13(c). It was different from that of the deterministic ML model (*D* = 0), wherein the firing frequency drastically increased from zero to the fixed value, as shown by the line with triangles in Fig 13(c). Similar result of firing frequency of type II excitability can be found in Figs 7–11 in a previous study [76].

### Changes in escape rate from stable focus with respect to noise intensity

**Fig 14:**
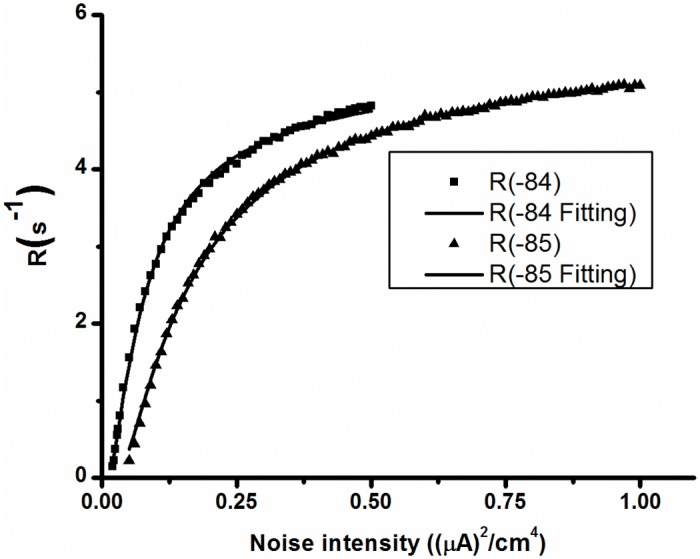
Changes of escape rate from the stable focus with respect to noise intensity. Square (triangle): simulation results when *V*_*K*_ = -84 mV (*V*_*K*_ = -85 mV); thin (bold) line: Fitting curve of simulation data when *V*_*K*_ = -84 mV (*V*_*K*_ = -85 mV) manifesting exponential function.

As mentioned above, the behavior of on-off firing pattern is noise-induced alternations between the stable focus and stable limit cycle. The generation of the burst is the passage from the stable focus to the limit cycle. The mean duration of the quiescent states is the mean first passage time. Correspondingly, the inverse mean duration of quiescent state is the escape rate (*R*) from the stable focus. As investigated in many previous studies of double potential wells, the relationship between the escape rate and noise intensity *D* is consistent with *R = Ae* ^*B/D*^, where *A* and *B* are related to the potential barrier between two potential wells or two stable behaviors. For example, the changes in *R* with respect to *D* were *R = 5*.*4215e* ^-0.06547/D^ for *V*_*K*_ = -84 mV (line with square) and *R = 5*.*8498e* ^-0.13737/D^ for *V*_*K*_ = -85 mV (line with triangle), as shown in Fig 14. The correlation coefficient between the simulation data and the fitting data was 0.99 for both *V*_*K*_ = -84 mV and *V*_*K*_ = -85 mV. The distinction of escape rate between *V*_*K*_ = -84 mV and *V*_*K*_ = -85 mV was induced by the difference in potential barrier, as the potential barrier between the coexisting attractors for *V*_*K*_ = -85 mV was larger than that for *V*_*K*_ = -84mV. The results showed that the on-off firing pattern was induced by noise.

### Coherence resonance in the stochastic ML model

The characteristic correlation time (*τ*_*c*_), the degree of coherence (β) and the amplitude of dominant peak of the power spectrum, which have been frequently used to describe CR [13], are employed to characterize the resonance that occurred in the stochastic ML model with *V*_*K*_ = -84 mV and *I* = 90.7 μA/cm^2^.

**Fig 15:**
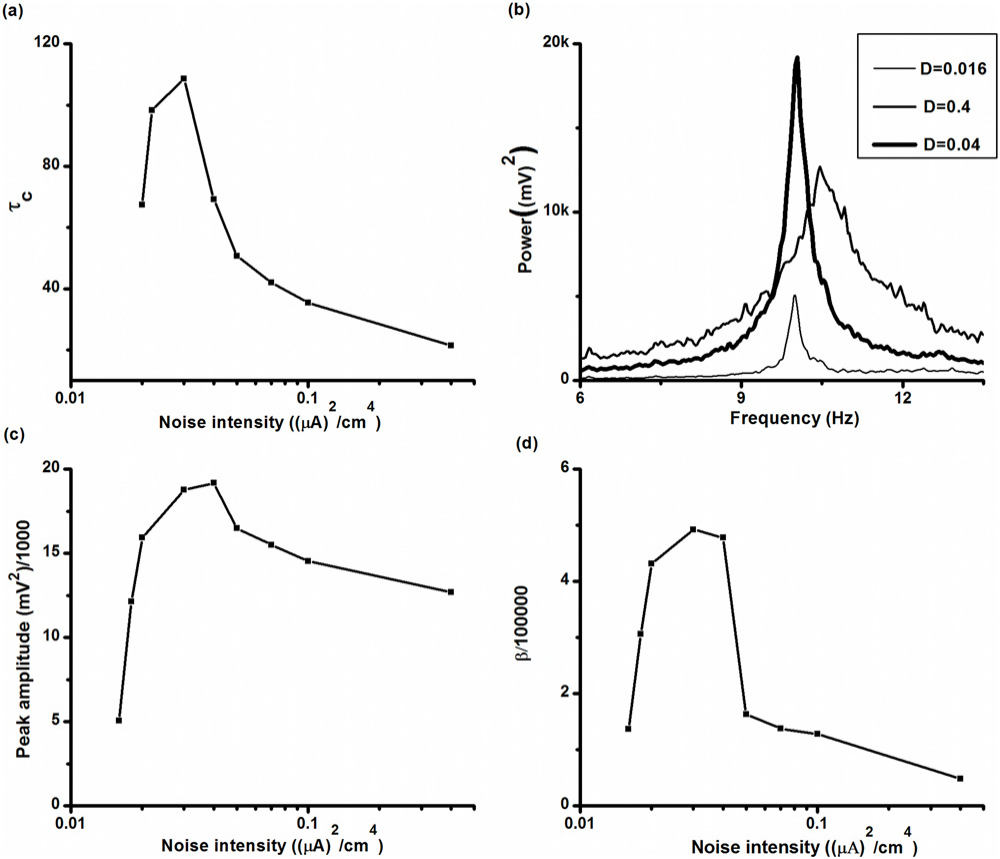
CR effect in the stochastic ML model with *V_K_* = -84 mV. (a) Characteristic correlation time *τ*_*c*_; (b) power spectrum of spike trains at different levels of noise intensity; thin (*D* = 0.016 (μA)^2^/cm^4^), bold (*D* = 0.04 (μA)^2^/cm^4^), and middle (*D* = 0.4 (μA)^2^/cm^4^) lines; (c) amplitude of dominant peak in the power spectrum; (d) degree of coherence (*β*).

The characteristic correlation time (*τ*_*C*_) is defined as$\tau_{c}=\int_{0}^{\infty }C^{2}(t)dt$, where *C*(*τ*) = $\frac{<\bar{V(t)}\bar{V(t+\tau )}>}{<\bar{V^{2}(t)}>}$ is autocorrelation function of firing train *V*(*t*), and$\bar{V(t)}=V(t)-<\bar{V(t)}>$. *τ*_*c*_ first increased to a maximum value and then decreased with increasing *D*, as shown in Fig 15(a), showing that CR was evoked.

The largest peak of the power spectrum of spike trains corresponding to three noise levels, *D* = 0.016 (μA)^2^/cm^4^, *D* = 0.04 (μA)^2^/cm^4^, and *D* = 0.4 (μA)^2^/cm^4^, are shown in Fig 15(b). β is defined with respect to the dominant spectral peak: β = *Hf*_*p*_/Δ*f*, where *f*_*p*_ is the frequency corresponding to the largest peak, *H* is the height of the peak at *f*_*p*_, and Δ*f* is the peak width corresponding to the height being as *H*/*e*. The higher and narrower the peak, the larger β. The amplitude of the largest peak of the power spectrum and β exhibited the signatures of CR. It first rose to a maximum and then decreased as *D* increased (Fig 15(c) and 15(d)).

In this paper, we provide a dimensionless indicator to characterize the CR effect of the on-off firing pattern. The indicator is based on the characteristics of the on-off firing pattern. The indicator is the ratio between two indicators of the on-off firing pattern. One of the indicators is the ratio of burst duration to the total duration of bursts and quiescent states, which is labeled as *R*_*b*_; the other is the CV of ISIs within the bursts, labeled as *CV*_*ISI*_. The indicator is the ratio of *R*_*b*_ to *CV*_*ISI*_.

**Fig 16:**
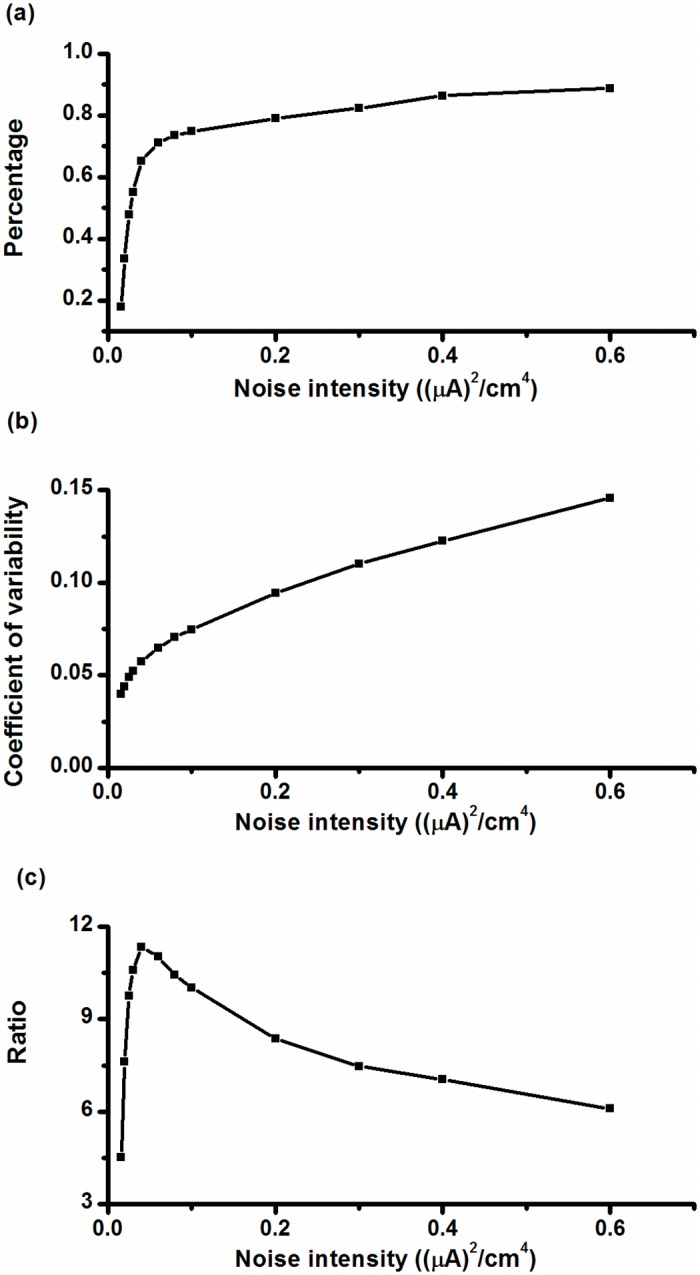
Changes in indicators with respect to noise intensity. (a) Relative number of burst durations within total durations of burst and quiescent state (*R*_*b*_); (b) CV of ISIs within bursts (*CV*_*ISI*_); (c) ratio of *R*_*b*_ to *CV*_*ISI*_.

With increasing *D*, *R*_*b*_ increased rapidly at first and then slowly, as shown in Fig 16(a), and *CV*_*ISI*_ increased slowly at first and then rapidly, as shown in Fig 16(b). The increase in *R*_*b*_ promoted the dominant peak in the power spectrum, and the increase in *CV*_*ISI*_ widened or shrank the dominant peak. *R*_*b*_ and *CV*_*ISI*_ had opposite effects on the power spectrum. When *D* was small, the effect of *R*_*b*_ on the power spectrum was dominant. When *D* became larger, the effect of *CV*_*ISI*_ became more dominant. Therefore, β and the height of dominant peak in the power spectrum increased initially and then decreased with increasing *D*. From this recognition, the ratio of *R*_*b*_ to *CV*_*ISI*_ was calculated to show the CR signature. It increased initially and then decayed with increasing *D*, as shown in Fig 16(c).

## Discussion

The dynamics of on-off firing patterns observed in three experimental models and in a theoretical stochastic ML model were investigated and compared. The on-off firing pattern alternated between bursts containing multiple period-1 spikes and quiescent states. The durations of both the bursts and quiescent states were long and had marked variations. The on-off firing pattern appeared between resting state and period-1 firing pattern when certain physiological parameters were changed. The ISI series of the on-off firing pattern was found to be stochastic. Based on the close match between the results of the experimental and theoretical models, the on-off firing pattern can be interpreted by stochastic effect on the bistable behavior near a sub-critical Hopf bifurcation. Noise was found to induce stochastic transitions between two coexisting attractors to form an on-off firing pattern alternating between bursts with multiple period-1 spikes and quiescent states over time.

The on-off firing pattern investigated in this paper was suggested to be stochastic, which is different from other viewpoints on the on-off firing pattern [30, 57]. For example, on-off firing pattern was suggested to be type III (elliptic) bursting or sub-Hopf/fold cycle bursting. However, no evidence suggested that type III bursting or sub-Hopf/fold cycle bursting were between resting state and period-1 firing, or that there were marked variations in durations of quiescent states and bursts. In addition, a firing pattern resembling the on-off firing pattern in appearance was simulated in a biophysical model [32], yet the bifurcation related to the firing pattern remains uncertain. The firing pattern obtained from the biophysical model should be compared with the on-off firing pattern obtained from the stochastic ML model in future.

**Fig 17:**
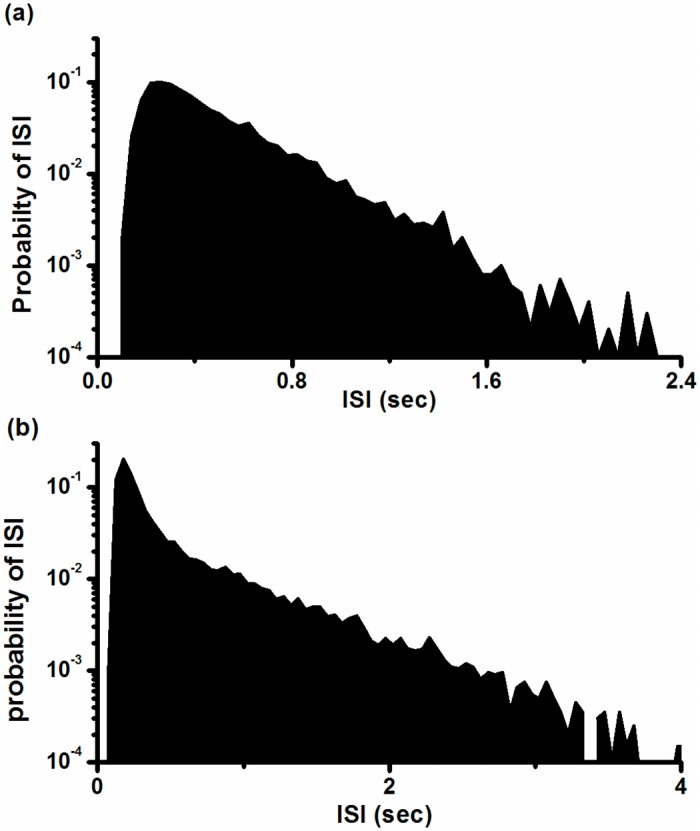
ISIH of stochastic firing pattern simulated in the stochastic ML model. (a) Corresponding to saddle-node bifurcation on an invariant cycle with *V*_*ca*_ = 20 mV, *V*_*K*_ = -84 mV, *ϕ* = 0.067, g_*Ca*_ = 4 mS/cm^2^, *V*_*3*_ = 12 mV, *V*_*4*_ = 17.4 mV, *I* = 39.6 μA/cm^2^, *D* = 0.1 (μA)^2^/cm^4^; (b) corresponding to saddle-node bifurcation with *V*_*2*_ = 42 mV, *V*_*3*_ = 12 mV, g_*K*_ = 10 mS/cm^2^, *V*_*K*_ = -84 mV, *I* = -57.3 μA/cm^2^, *D* = 0.05 (μA)^2^/cm^4^. Other parameter values were the same as those provided in the introduction of the ML model.

The on-off firing pattern manifested characteristics different from noise-induced stochastic firing patterns near other three kinds of bifurcation. Integer multiple firing pattern simulated near a super-critical Hopf bifurcation point manifested more single spikes than the on-off firing pattern, showing more frequent transitions between the spikes and the quiescent states. The reason may be that in the deterministic model, both period-1 firing and resting state are stable near the sub-critical Hopf bifurcation point, whereas only one attractor is stable near the super-critical Hopf bifurcation point. The stochastic firing patterns corresponding to both Hopf bifurcations had discrete distributions in ISIH and a basic ISI, which corresponded to the fixed frequency of type II excitability. Noise-induced stochastic firing patterns near the saddle-node bifurcation on an invariant cycle or near the saddle-node bifurcation of equilibrium point exhibited a continuous distribution of ISIs in unimodal ISIH, as shown in Fig 17. The four different types of ISIH can be used to distinguish types of bifurcations.

Further, based on the relationship between ISIH and bifurcation types, the four different types of ISIH can also be used to discriminate types of neural excitability [77–90]. As reported in some previous studies, type I excitability was often associated with saddle-node bifurcation on an invariant circle, and type II excitability was related to Hopf bifurcation except for a special condition of saddle-node (off limit cycle) bifurcation [78, 79]. In addition, two indicators that have been used to discriminate types of excitability, the firing frequency and CV of ISIs [91], should be reconsidered. In general, as the stimulus intensity or biological parameter varied, the firing frequency varied from zero for type I excitability and from a non-zero value for type II excitability. However, the firing frequency varied from zero for type II excitability in experiments and in theoretical models with noise, as shown in Fig 13(c), suggesting that firing frequency may lose effectiveness to discriminate neural excitability types due to stochastic effects of noise. In previous studies [81, 82], a firing pattern with a CV value larger than 0.6 was related to type I excitability, while a firing pattern with a CV lower than 0.3 was related to type II excitability. In the present study, both the on-off firing pattern and the integer multiple firing pattern had a CV larger than 0.6, suggesting that a larger CV value is not unique for discriminating type I and II excitabilities.

When this stochastic on-off firing was present in the neuronal system, a CR effect was induced. It indicated that noise might play important roles in the generation of the on-off firing patterns and in the implementation of physiological functions in depressor nerves and CA1 neurons. Noise has been confirmed to play an important role in sensory encoding in crayfish, cricket, dogfish, and paddlefish by application of CR or SR [1, 13, 15, 92–94]. In addition, changes in the escape rate from the stable focus with increasing noise intensity were found to be exponential, which was consistent with the rules of stochastic dynamics [95].

The on-off firing pattern has been observed in many different kinds of nervous systems by adjusting ion concentration, ion channel conductance, drug, depolarization current, noise, or high frequency stimulation [26–56], suggesting that the on-off firing pattern is common. For example, the on-off firing pattern was observed in most examples of the CCI model and the CCD model under control condition [28, 29]. Period-1 firing can be changed to the on-off firing pattern at first and then to resting state by application of riluzole or gabapentin [28]. Riluzole has been used as a relatively specific persistent sodium channel blocker and clinically in the treatment of several neurological disorders, including amyotrophic lateral sclerosis and epilepsy. Gabapentin has been shown to be effective in suppressing pain in animal models of neuropathic pain and in chronic pain patients [29]. These results, therefore, suggest that on-off firing pattern may participate in pathological pain. In addition, on-off firing pattern was suggested to participate in the generation of beta or gamma rhythms and in cognition [26]. More studies are needed to elucidate the dynamics of the on-off firing pattern in different neural tissues.
